# Apnoea of Prematurity and Neurodevelopmental Outcomes: Current Understanding and Future Prospects for Research

**DOI:** 10.3389/fped.2021.755677

**Published:** 2021-10-25

**Authors:** Max Williamson, Ravi Poorun, Caroline Hartley

**Affiliations:** ^1^Department of Paediatrics, University of Oxford, Oxford, United Kingdom; ^2^Department of Paediatrics, Royal Devon and Exeter NHS Foundation Trust, Exeter, United Kingdom

**Keywords:** apnoea of prematurity, neurodevelopment, brain, preterm, neonate, caffeine

## Abstract

Infants who are born prematurely are at significant risk of apnoea. In addition to the short-term consequences such as hypoxia, apnoea of prematurity has been associated with long-term morbidity, including poor neurodevelopmental outcomes. Clinical trials have illustrated the importance of methylxanthine drugs, in particular caffeine, in reducing the risk of long term adverse neurodevelopmental outcomes. However, the extent to which apnoea is causative of this secondary neurodevelopmental delay or is just associated in a background of other sequelae of prematurity remains unclear. In this review, we first discuss the pathophysiology of apnoea of prematurity, previous studies investigating the relationship between apnoea and neurodevelopmental delay, and treatment of apnoea with caffeine therapy. We propose a need for better methods of measuring apnoea, along with improved understanding of the neonatal brain's response to consequent hypoxia. Only then can we start to disentangle the effects of apnoea on neurodevelopment in preterm infants. Moreover, by better identifying those infants who are at risk of apnoea, and neurodevelopmental delay, we can work toward a risk stratification system for these infants that is clinically actionable, for example, with doses of caffeine tailored to the individual. Optimising treatment of apnoea for individual infants will improve neonatal care and long-term outcomes for this population.

## Introduction

Apnoea of Prematurity (AOP) is frequently defined as a pause in breathing lasting more than 20 s, or more than 10 s with accompanying bradycardia and/ or oxygen desaturations in an infant born before 37 weeks' gestation (1–3). AOP is one of the most common diagnoses in the Neonatal Intensive Care Unit (NICU) (3), affecting almost half of infants before 32 weeks' gestation and nearly all whose birthweight is below 1000 g (4). Along with acute morbidities such as cyanosis and chronic intermittent hypoxia, a key consideration for these infants is whether AOP also influences their health later in life. This is particularly the case with regards to the central nervous system, which is highly sensitive to hypoxia. Children born prematurely are much more likely to suffer from poor neurodevelopmental outcomes than those born at term (5–9), and some studies have suggested that this could in part be related to the frequency of apnoeic events and desaturations they experience in early life (10, 11). Here we will review the existing literature with regards to the mechanisms of AOP and its possible role in long-term neurodevelopmental disorders in premature infants. We then discuss key research questions in this area moving forward and approaches to investigate these problems.

## Central and Peripheral Mechanisms of Apnoea of Prematurity

Apnoeas are classified as central or obstructive, in that they either derive from insufficient drive from the respiratory centres of the brain, or that the airway itself is obstructed. “Mixed” apnoeas are a combination of the two. AOP is thought to be a physiological consequence of immaturity, with insufficient central respiratory drive to maintain ventilation across the lung (11, 12). Subsequent reductions in airway tone frequently make these apnoeas mixed (13).

Central control of breathing pertains to the role of the central nervous system in regulating rhythmicity and expansion of the chest in respiration. Normal inspiratory rhythm at rest is directed by the medullary dorsal respiratory group of neurons, with the pneumotaxic centre of the pons acting to slow inspiration. Expiration is the result of reduced dorsal respiratory group output and elastic recoil of the chest wall. The ventral respiratory group of the medulla is primarily involved in active expiration and tachypnoea, usually in exercise, in response to stimulation by peripheral chemoreceptors, and the pre-Bötzinger complex within the ventral respiratory group is an intrinsic site of rhythmogenesis (i.e., the process of breath generation) (14–16).

*In utero*, foetal breathing movements are only intermittent, and work to facilitate lung bud growth through mechanical stretching, rather than for gas exchange (17, 18). As such, these respiratory control regions of the brain predominantly mature postnatally and are unable to maintain stable breathing patterns or respond effectively to stimulation from chemoreceptors, lung stretch receptors, or other regions of the brain beforehand (19). This physiological immaturity is presumed to be the primary cause of apnoea in infants born prematurely (20). For example, central response to carbon dioxide (CO_2_) in the medulla is the primary controller of respiratory drive (21), but hypercapnic responses can destabilise tidal breathing rhythm in premature infants. In those born at term, hypercapnia induces respiratory compensation through increases in tidal volume and respiratory rate; in prematurely born infants, hypercapnia induces an increase in tidal volume but not respiratory rate, resulting in bradypnoea and desaturations (22). This also results in an increased apnoeic threshold of CO_2_ that is close to tidal partial pressures of CO_2_, giving premature infants a high propensity for apnoeic events (23, 24).

**Figure 1:**
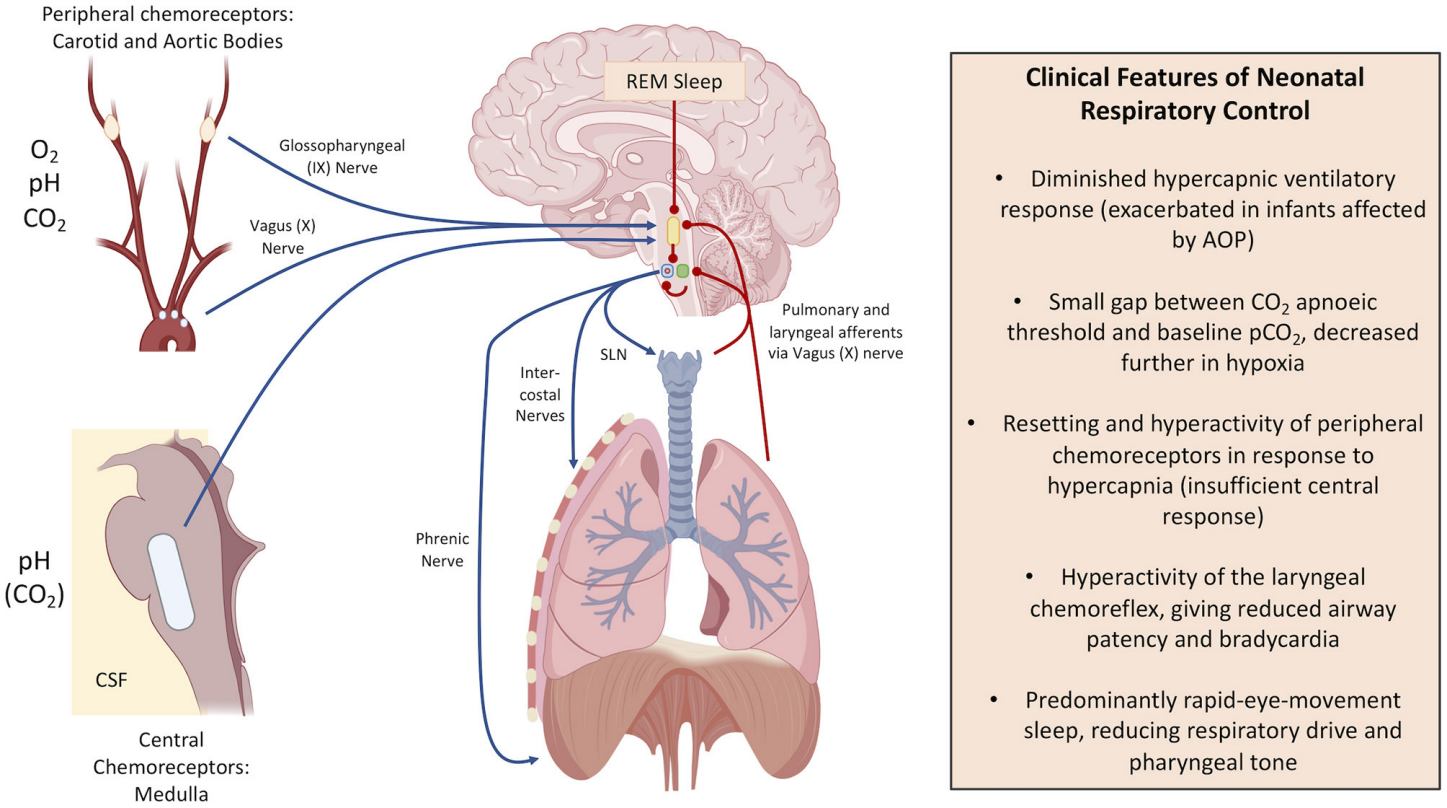
Respiratory control mechanisms and the differences in infancy. Blue arrow: positive stimulation (encourages ventilation). Red lines: negative stimulation (depresses ventilation). In the brain: the yellow shape indicates pontine respiratory centre, the green circle indicates the dorsal respiratory group, the blue circle indicates the ventral respiratory group, and the red dot illustrates the pre-Bötzinger Complex. SLN is the Superior Laryngeal Nerve. This figure was created using BioRender (https://Biorender.com).

Along with the central respiratory centres of the brain, several other mechanisms play a role in AOP, summarised in Figure 1. Dramatic shifts in the physiology of the peripheral chemoreceptors follow with premature birth. Peripheral chemoreceptors of the carotid and aortic bodies respond to low arterial partial pressures of oxygen and high partial pressures of carbon dioxide *via* the hypoglossal (IX) and vagus (X) nerves, respectively. This normally triggers homeostatic breathing responses such as an increase in ventilation in response to hypercapnia through greater stimulation of the ventral respiratory group (21). Peripheral receptors are silenced at birth in response to changes in oxygen saturations with the baby's first breath, with their functions then progressively restored in infants born at term so that they can respond appropriately to changes in blood gases (25–28). Chronic intermittent hypoxia gives rise to hyperactive peripheral chemoreceptor responses, especially in those who suffer from AOP (25). This contributes to the hypoventilation in response to hypercapnia in infants with AOP, and can give rise to periodic breathing that is characteristic of unstable respiratory control (29, 30).

Spontaneous neck flexion is known to precipitate apnoeic events, and it is recommended that all babies who suffer from AOP are positioned prone to mitigate this (31). The airway requires tonic stimulation of the neck muscles to remain open. Moreover, stretch and irritant receptors in the lungs and trachea can initiate the cough reflex, which can bring about bradypnoea and apnoea by destabilising central rhythmogenesis. Fleming et al. (32) showed that in intubated premature infants whose irritant receptors were stimulated clinically, only one of the 18 babies born before 35 weeks' gestation showed a “mature” bronchial response, with all others showing sustained bradypnoea or apnoea (32). This was mirrored by later work which suggested a correlation between acid reflux, consequent hyperactivity of the laryngeal chemoreflex, and apnoeic events (33–35), although the association remains controversial (36, 37).

Sleep also has a profound effect on respiratory control. Premature infants predominantly show rapid-eye-movement (REM, otherwise known as active) sleep (38, 39), which decreases central respiratory control and reduces tonic contraction of pharyngeal muscles, resulting in obstruction and apnoea in premature infants with hypercompliant, low-calibre airways (11, 39–41).

## The Long-Term Consequences of AOP

Apnoea in preterm infants is frequently associated with bradycardia and oxygen desaturations. These events are precipitated by the arterial chemoreflex in response to hypoxaemia (42–44), and the lack of sinus arrythmia (ordinarily initiated by the pulmonary inspiratory reflex) sustains the bradycardia and desaturations over time (11, 45, 46). Moreover, arterial partial pressures of oxygen are typically maintained at 50–80 mmHg in premature infants (42, 43); the oxygen-haemoglobin dissociation curve is much steeper at these values than in the higher pressures of the adult making premature infants more sensitive to desaturations with apnoea (23).

**Figure 2:**
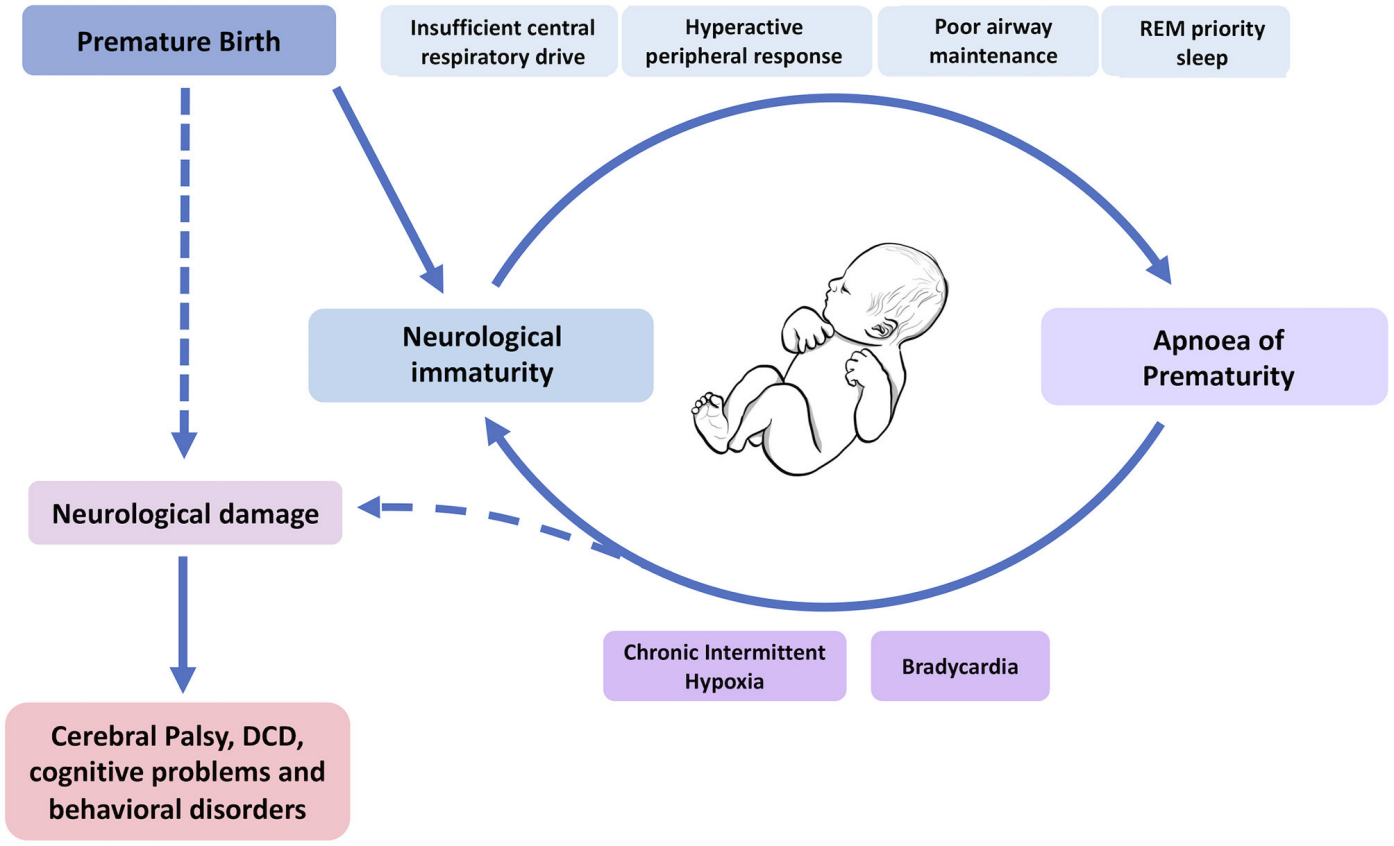
Which comes first, the immaturity or the apnoea? A model of the interplay between the causes (top) and consequences (bottom) of apnoea of prematurity and respiratory immaturity in neonates, and how this might lead to neurodevelopmental impairment. DCD, developmental coordination disorder. Dashed lines indicate mixed causes between the sequelae of AOP and prematurity itself on neurological damage in these infants. This figure was created using BioRender (https://Biorender.com).

AOP ordinarily resolves with time and increasing maturity of the infant's respiratory systems. However, apnoea and the consequential chronic intermittent hypoxia, may have long-term effects including increased risk of infant mortality (47), neurodevelopmental impairment (10, 48) and retinopathy of prematurity (ROP) (49, 50). Janvier et al. (10) suggested a link between days of hospitalisation with apnoea and worse neurodevelopmental outcomes at 3 years follow-up (including visual and hearing impairment) that was independent of other variables including sex and overall time spent in hospital. Similarly, Pillekamp et al. observed an association between both delayed resolution of apnoea and higher daily apnoea with poorer neurodevelopmental outcome at 13 months of age (48). Moreover, Poets et al. found that prolonged hypoxemic episodes (oxygen saturation of <80% for at least 10 s, which may or may not be related to apnoea) were associated with increased risk of death or disability at 18 months of age in a large study of 972 infants (47). However, these studies identify statistical correlations which are not necessarily causative. Further, we must remember that the AOP exists in the background of prematurity itself, and there are numerous other factors related to prematurity that have been associated with long-term negative consequences (5, 6, 51–54). So, whilst we now have a better understanding of AOP itself, the clinical reality of prematurity makes research in this field complex, and full knowledge of the implications of our interventions on neurodevelopment will not be known for years after these infants are treated. Further research is needed to understand the interplay between neurological immaturity at birth, the impact of prematurity, AOP and neurodevelopmental outcomes (Figure 2).

## Caffeine for the Management of AOP and Its Relationship With Long-Term Effects

Whilst mechanical respiratory support and non-invasive ventilation such as continuous positive airway pressure (CPAP) play an important role in supporting infants with AOP, pharmacological interventions are now a mainstay of treatment (3). Methylxanthines, in particular caffeine, are the most commonly used pharmacological intervention for the treatment of AOP. Other interventions, such as doxapram, are also used as an adjunct therapy in some NICUs in individuals where caffeine treatment is not effective (55, 56).

Methylxanthines began to be used for the treatment of AOP in the 1970's, following the first observational study by Kuzemko and Paala in 1973 showing a complete prevention of cyanotic attacks in nine of 10 apnoeic neonates administered with aminophylline (57). The first randomised clinical trial of this drug showed a reduction of apnoeic events after 8 h in all of the 14 participants (57, 58). Of all the methylxanthines, caffeine citrate is often now the drug of choice due to its longer half-life, higher therapeutic index, and decreased need for drug-level monitoring (3). It is currently the third most commonly prescribed drug in European NICUs (59). Methylxanthines structurally mimic nucleosides, blocking neuronal adenosine receptors A_1_ and A_2A_, with A_1_ being the primary inhibitory receptor of postsynaptic neuron activation in response to hypoxic stress (60, 61). By inhibiting these receptors, methylxanthines increase central chemosensitivity to CO_2_ and generate a more active respiratory response (62).

Most of the early data on the use of methylxanthines derived from small studies with limited follow-up, and showed conflicting results (55, 63–66). In 2006 an international placebo-controlled trial investigated caffeine use for AOP-The Caffeine for Apnoea of Prematurity (CAP) trial (67)-began to publish results advocating the short and long-term efficacy of caffeine. The CAP trial randomised 2006 premature infants with AOP into a caffeine intervention group vs. a saline control group and has followed them up for 11 years to track neurodevelopmental outcomes. Whilst there was no difference in perinatal mortality between the groups, there was a significant reduction in bronchopulmonary dysplasia at discharge in the caffeine-treated group, and the length of time on all forms of ventilatory support was reduced by ~1 week (67). Eighteen months after birth, children treated with caffeine were less likely to suffer from cerebral palsy or neurodevelopmental delay than the control group. *Post-hoc* analysis revealed that post-menstrual age of earlier ventilatory support removal explained nearly half (49%) of the variability of these data (68), arguing for the hypothesis that prolonged ventilatory support comes with long term toxicity for neonates, one that has been supported by more recent evidence of neurotoxicity with mechanical ventilation both in preterm infants and animal models (69–71). Equally, these infants were also less likely to be exposed to further pharmacological interventions, such as corticosteroids or non-steroidal anti-inflammatories, and less likely to require surgical or pharmacological closure of patent ductus arteriosus (67), each of which carry their own risks in terms of neurodevelopment (72, 73). Follow-up at 5 (74) and 11 (75) years did not demonstrate significant behavioural or cognitive differences in the caffeine-treated group compared to controls, but found a reduction in children with developmental coordination disorder (DCD) at 5 years (76). Given that DCD, a generalised motor dyspraxia without neuromuscular involvement that significantly affects daily activities (77), has been shown to reduce self-reported physical well-being, financial resources and positive school environment (78), it is clear that mitigating this morbidity can significantly impact on the quality of life of these infants (76, 79–82).

Thus, these results make a strong case for caffeine improving the long-term as well as the short-term outcomes for premature-born children with AOP. As a result, caffeine for AOP has now been lauded as one of neonatology's greatest success stories (83, 84). Nevertheless, many questions remain about its use. For example, what is the optimal dosing regimen, the optimal time and duration for treatment, and should therapeutic drug monitoring be used (85)? Some studies have suggested detrimental effects with increased caffeine dose, including a pilot study in 2012 which found increased incidence of cerebellar haemorrhage with a high dose caffeine regime vs. standard dose that persisted after correcting for confounding factors such as gestational age and vasopressor exposure (86). Another observational prospective study demonstrated an association between high serum caffeine levels and pro-inflammatory cytokine profiles (87). So, caffeine is no panacea for AOP. As with all medical interventions, it comes with risks that we are still yet to fully delineate. Moreover, nearly 20% of the caffeine-treated cohort in the CAP trial still scored poorly in motor function tests, with no amelioration of academic or behavioural performance (75): the story must not end here.

## Where Next for Apnoea of Prematurity?

There has long been a recognition in the literature that AOP is variable in its penetrance, with the disorder carrying on into term age for some of the more prematurely born infants, whilst not affecting others (23, 46). There is also wide variation in individual infants' responses to caffeine therapy (88); some infants respond well to treatment whilst others continue to have high numbers of apnoeic episodes and require higher doses of caffeine. Moreover, in most infants, caffeine therapy can be stopped at around 34 weeks postmenstrual age without problems, but ~10% of infants will develop episodes of apnoea after stopping treatment requiring the reintroduction of caffeine therapy (89). Key questions for the future will be in risk stratification of babies with AOP and providing individualised treatment options. Can we predict which infants are most at risk of experiencing apnoea and when? Which of these will be improved with dose-stratified caffeine treatment? Which infants with AOP are most at risk of long-term neurodevelopmental deficits? To address these questions, we propose that we first need (1) improvements in AOP measurement in the NICU and (2) a better understanding of the impact of apnoea on brain development in preterm infants.

## Improvements in AOP Measurement

Respiration, and consequently apnoea, is frequently measured in the NICU using the impedance pneumograph. This measures the electrical impedance of the chest *via* electrocardiogram electrodes, giving us a measurement of chest wall movement. Alternatively, respiratory dynamics can be measured routinely using the Graseby Capsule, a small pneumotaxic device designed to measure the movement of the xiphisternum with each breath (90), or using respiratory inductance plethysmography for example. However, these methods can be sensitive to non-respiratory related movements and cardiac activity (91, 92), and purely obstructive apnoeas cannot be identified using these techniques.

To further define the role of AOP in neurodevelopmental outcome, and to better treat apnoea, a more accurate and reliable measurement of AOP is required. Lee et al. (91) developed a new method for the detection of apnoea from the impedance pneumograph by first removing cardiac interference. Our group recently built on this work and developed a new method to identify inter-breath intervals and apnoeas in infants which included an automated classifier to distinguish between periods of true apnoea and signal which is low amplitude due to artefacts or poor electrode placement (93). Using these methods could improve apnoea detection and consequently our understanding of apnoea in preterm infants; 74% of apnoeic events across 276 infants identified using the algorithm of Lee et al. were not documented in clinical notes (94). Similarly, in our study we found that 88% of apnoeas were missed in clinical notes (93).

An improved understanding of infant respiratory dynamics would greatly supplement recent advances in ventilation technology, in particular the use of Neurally Adjusted Ventilation Assistance (NAVA) (95). NAVA allows for a more precise control of ventilatory support directly in response to changes in the infant's own respiratory drive and can provide backup ventilation during periods of apnoea (96, 97). In a single-centre retrospective pilot study of 17 infants with AOP, switching from traditional CPAP to non-invasive NAVA demonstrated a significant reduction in apnoeic events (98). A similar study of 108 very-low-birth-weight infants with AOP comparing NAVA to nasal intermittent positive pressure ventilation identified less bradycardic events in the former compared to the latter (99). Questions remain regarding the scalability of this technology to the wider population and the need for large sample long-term follow-up studies (97), but equally, NAVA could represent an effective way of both measuring and responding to apnoeic events as they occur. Moreover, automated oxygen titration systems are beginning to be used in some NICUs following recent work highlighting their safety (100). The impact of these systems on AOP dynamics warrants investigation.

With better measurement of AOP and respiratory dynamics we will be able to develop techniques to improve our treatment for apnoea. Computational techniques, such as machine learning algorithms, have the potential to predict apnoeic and hypoxemic events in individual infants, leading to earlier interventions (101). Optimising caffeine dosing regimen and tailoring pharmacological interventions, including doxapram (102), for individual infants could be achieved through accurate measurement of respiratory dynamics and will shift the balance toward efficacy and away from harm. Pharmacokinetics and pharmacodynamics will likely change with postnatal age, for example due to changes in hepatic enzymes and renal function (103), and so continuous monitoring of respiratory dynamics and adaptive treatment will be important. Moreover, we should consider using information on respiratory dynamics in conjunction with pharmacogenetic approaches, particularly given the wide metabolic variation across individuals of caffeine metabolism in the liver (104). Our goal for the future should be to establish a dosing regimen of both pharmacological interventions and ventilatory assistance for infants that is stratified and targeted based on the risk of developing apnoea (92).

## Understanding the Impact of Apnoea on Brain Development in Preterm Infants

Studies to date which have investigated a possible role of apnoea on later life neurodevelopmental outcomes have shown a correlative rather than causative relationship (10, 11, 48, 105, 106). To take the extreme, it could be that infants with poorer brain function at birth are more likely to have apnoeas and concurrently have poorer neurodevelopmental outcomes later in life, with the episodes of apnoea themselves not affecting brain development. On the other hand, given the resultant hypoxia, it is plausible that episodes of apnoea do have an impact on neurodevelopment. Understanding the long-term impact that AOP has on neurodevelopment requires direct investigation of the effects of apnoea on infant brain structure and function.

Numerous MRI studies have investigated differences in the brain structure in preterm infants as they develop (107–110). However, to our knowledge, the only investigation to consider the relationship with AOP so far is the volumetric MRI studies of 70 CAP trial cohort patients, which found no substantial differences in gross brain volume or white matter distribution, except for a small decrease in growth of the corpus callosum in the caffeine treated group compared with the placebo controls (111). This finding is interesting given a previous diffusion-weighted MRI study which found changes in the axial diffusivity of the parietal aspects of the corpus callosum in children with developmental coordination disorder (110). Longitudinal follow up with neuroimaging of infants with AOP will be an important path to deciphering how the brain is impacted by AOP and how infants respond to treatment. However, studies of brain structure will also necessarily be limited to snap-shot images of particular timepoints. To develop a clearer theory of the relationship between AOP and neurodevelopment, we must also examine the impact of apnoea on brain function.

For this purpose, techniques such as electroencephalography (EEG) can be used at the cot-side and have excellent temporal resolution, enabling the investigation of brain function before, during and after apnoeic episodes. Whilst a number of EEG studies have described seizure-related apnoeic episodes in infants, changes in the EEG related to non-seizure apnoeic episodes have not been described in detail (112–114). Low et al. found that apnoeic events precipitated EEG suppression in a case report of a single infant (112). Whilst the authors suggest that EEG suppression may be neuroprotective, it is also conceivable that frequent interruptions in brain activity could be disruptive to neurodevelopment during critical periods (115). Animal studies have also observed similar effects, with a study in piglets demonstrating EEG suppression within 30 s of the start of episodes of apnoea induced through stimulation of the superior laryngeal nerves (116). How changes in the EEG with apnoea relate to factors such as the age of the infant and the degree of hypoxia, as well as individual differences across infants, is yet to be explored. Understanding how and when apnoeas alter brain function will shed light on the neurological pathophysiology of AOP and could aid stratification of infants at risk of neurodevelopmental problems.

## Conclusion

There have been vast improvements in the understanding and treatment of AOP over the last 40 years, and the CAP trial is illustrative of the positive impact this understanding can have on the neurodevelopmental outcomes of children born prematurely. However, questions remain about the exact role of AOP in contributing to the worse neurodevelopmental outcomes faced by premature-born babies. To better understand the impact that apnoea has on the long-term outcomes of preterm infants, we must work toward more sophisticated measurements of apnoea and investigate further the brain's response to hypoxia. Improved measurement of AOP and respiratory dynamics in infants will allow us to stratify infants based on the risk of developing AOP and the risk of poor neurodevelopmental outcomes. Doses of pharmacological interventions and ventilatory support could then be tailored to the individual, optimising the balance between the benefits and risks of these interventions. With these measures in place, we may be able to move closer to a scenario where no infant born prematurely is at risk of adverse neurodevelopmental outcomes because of AOP.

## Author Contributions

MW wrote the first draft of the manuscript, with supervision from CH. All authors critically reviewed and revised the manuscript.

## Funding

CH is funded by the Wellcome Trust and Royal Society through a Sir Henry Dale Fellowship (Grant Number: 213486/Z/18/Z).

## Conflict of Interest

The authors declare that the research was conducted in the absence of any commercial or financial relationships that could be construed as a potential conflict of interest.

## Publisher's Note

All claims expressed in this article are solely those of the authors and do not necessarily represent those of their affiliated organizations, or those of the publisher, the editors and the reviewers. Any product that may be evaluated in this article, or claim that may be made by its manufacturer, is not guaranteed or endorsed by the publisher.
